# Architectural Designs in Maddox Street

**Published:** 1913-02-01

**Authors:** 


### ARCHITECTURAL DESIGNS IN MADDOX STREET.

### Some Tendencies of Institutional Planning.

At the Architectural Association's premises, Westminster, the exhibition of designs and details by the late Mr. Norman Shaw, R.A., whose recent death has focussed public interest in his works, has been visited by persons of all ranks and professions. It was with a desire to find out whether this master-mind had ever been applied to the subject of hospital design that we visited the exhibition, but unfortunately could not find any detail whatever on this important subject. Another exhibition at the Royal Institute of British Architects in Maddox Street, W., is the antithesis to the Shaw exhibition. The latter contains the work of a man who died full of years, esteemed as the doyen of his profession, and loved by all; whereas the former consists of works by students on the threshold of their careers. It is interesting to the layman to observe that, however young the architect may be, he has fantasies and noble ideas, which his draughtsmanship sometimes fails to rise to. The subjects include railway terminals, Royal Palace façades, and other subjects in which the student is free to revel without any restriction such as cost to hamper him. The subject, however, of particular interest to Hospital readers is the Saxon Snell £60 prize for a design of a home for 150 consumptives; and it is regrettable that, whereas some of the subjects tempted no less than twenty-nine competitors, only three designs were submitted in this competition. This may be because the design for a façade is the logical educational development of the student's training; whereas the subject of hospital design is a specialty, and many architects know nothing of the subject until they are called upon in a professional capacity to design an institution. It was probably this fact which prompted the late Mr. Snell to provide the studentship.

The conditions provide for a site with a main roadway along its northern boundary, and with a fall in the land towards the south; in other words, an ideal site.

Mr. L. G. Pearson provides a three-storey building for patients, with a south aspect; and axially to the north a three-storey administrative block. To the north of that is the one-storey recreation and dining-hall block, and beyond the kitchen and boiler-house, flanked on the east by laundry and on the west by stables. It seems that coherence has been lost in the desire to place the administrative buildings with their axial lines due north and south. His scale detail shows a room 14 feet by 10 feet and 9 feet high, opening off a corridor on the north side, and having a southern prospect through casement windows. A feature of the elevation is external louvred sliding shutters, an original detail which might be further developed to great advantage. Sound-proof hollow partitions separate the bedrooms from one another, and the detail shows all the furniture; but the student should remember that this class of building is furnished in an essentially different manner to the modern hotel. It is also regrettable that no fireplace is provided in the bedrooms; a cosy fire is not only cheering to the invalid, but is a perfect form of ventilation.

Mr. Jefferies, in a careful set of drawings, shows also a three-storey bedroom block. The main approach from the north leads direct to the entrance hall, which gives place on the west to administrative quarters and on the east to dining and recreation halls, with a kitchen block to the north. The detail shows a bedroom, 15 feet by 12 feet by 14 feet high, heated by an anthracite stove; and French folding casements give access to a balcony which can be separated into bays by screens arranged to fold back against the wall.

These two designs are based on the Continental method of planning several storeyed buildings; but the tendency of modern English planning is towards the open-verandah bungalow type of building, one storey high, simply arranged, and served by a one or two storeyed administrative block: in fact, a more or less developed type of isolation hospital. Such a type of plan gives perfect air circulation, and makes simple the segregation of sexes and stages of convalescence; and permits of easy expansion without affecting existing structures. Probably this tendency of modern planning influenced the assessors in awarding the place of honour to Mr. Vincent Hooper, though his method of draughtsmanship is in any case commendable for its originality. His approach is roughly 150 feet east of the axial line of the site, and leads directly to a three-storeyed administrative block. On the axial line is placed the kitchen and dining-rooms; and to the west, more or less balancing the administrative block, is a two-storey hospital block, presumably for acute cases. Some 200 feet to the south are placed four blocks of cottages for ambulant patients, providing 100 beds in all.

A Byzantine chapel is placed centrally between the hospital building and the cottages. The boiler-house is placed right away to the east, which would seem to give an unnecessarily long drive for the heating and hot-water system. The scale detail shows a typical room 10 feet high and 12 feet by 10 feet. The system of heating is by radiator, and a tile dado is shown all round, except that portion of the wall occupied by casement windows. The system of drainage shows a tank system, and the effluent is carried to a convenient stream.

The three designs are interesting to all concerned with consumptive problems, and it is lamentable that the responses have not been more numerous. The exhibition remains open until February 3, and at 8 P.M. the President of the Royal Institute will address the students and present the prizes, and thereafter Mr. Curtis Green will give a criticism of the designs submitted.

